# Muc16 contributes to protection against invasive pneumococcal infection originating from nasal colonization

**DOI:** 10.1128/spectrum.00824-26

**Published:** 2026-06-26

**Authors:** Daichi Murakami, Tatsuya Shiga, Hideki Sakatani, Takuro Iyo, Hiroki Kuwazoe, Kazuya Mizobata, Ryo Ueda, Shizuya Saika, Muneki Hotomi, Masamitsu Kono

**Affiliations:** 1 Department of Otorhinolaryngology-Head and Neck Surgery, Wakayama Medical University 13145, Wakayama, Japan; 2 Department of Ophthalmology, Wakayama Medical University 13145, Wakayama, Japan; The Ohio State University College of Dentistry, Columbus, Ohio, USA

**Keywords:** *Streptococcus pneumoniae*, invasive pneumococcal disease, Muc16, mucosal disruption, cigarette smoke

## Abstract

*Streptococcus pneumoniae* asymptomatically colonizes upper respiratory mucosa and invades into deeper tissue through mucosal membrane. Invasive pneumococcal disease has a significantly high rate of mortality, making its control an urgent concern. Mucin is a defensive system against respiratory infection. However, the role of transmembrane mucin against the development of invasive infection has been unclear. In this study, we demonstrate the roles of Muc16 on invasive pneumococcal disease. Muc16 knockout mice had significantly increased bacterial loads in the lungs and brain after nasal pneumococcal challenge, whereas bacterial loads in the nasal cavity had no difference. Under cigarette smoke extract (CSE)-pretreated conditions, they showed increased bacterial loads in the blood with the elevation of serum IL-6 level and a significant increase in the mortality rate. Infiltration of polymorphonuclear leukocytes was suppressed by deficiency of Muc16. The proportion of mucosal disruption in the olfactory epithelium was significantly increased by deficiency of Muc16, and the expression of tight junction molecule ZO-1 was inhibited in CSE-pretreated Muc16 knockout mice. Muc16 is, thus, considered to be essential for preserving the defensive system against pneumococcal translocation from nasal mucosa by regulating inflammatory cell infiltration and mucosal barrier integrity in the upper respiratory tract. MUC16 could be a new target in preventive strategy against pneumococcal infection.

**IMPORTANCE:**

*Streptococcus pneumoniae* commonly colonizes the upper respiratory tract without causing symptoms but can sometimes invade deeper tissues and lead to life-threatening diseases, such as pneumonia, bacteremia, and meningitis. The mechanisms that prevent bacteria from spreading beyond the nasal mucosa are still incompletely understood. In this study, we demonstrate that the transmembrane mucin MUC16 plays an important role in protecting against invasive pneumococcal infection. Using a mouse model, we show that loss of Muc16 does not alter nasal colonization but increases bacterial dissemination to systemic organs and worsens survival, particularly under cigarette smoke exposure. Our findings suggest that MUC16 contributes to mucosal defense by maintaining epithelial barrier integrity and supporting neutrophil recruitment at the nasal surface. These results highlight the importance of transmembrane mucins in preventing bacterial invasion and provide new insight into how disruption of mucosal barriers may promote invasive pneumococcal disease.

## INTRODUCTION

*Streptococcus pneumoniae* can colonize the nasopharynx asymptomatically, and under certain conditions, it has the potential to cause severe invasive infections, such as pneumonia, bacteremia, and meningitis. The subsequent development of invasive pneumococcal disease (IPD) from nasopharyngeal colonization is multifactorial. To establish colonization and facilitate subsequent invasion, *S. pneumoniae* must overcome the mucosal glycocalyx and disrupt the epithelial barrier of the upper respiratory tract (1–4). IPD is associated with significant morbidity and mortality, so we suggest it is of urgent clinical importance to understand the factors that facilitate transition from the nose to the bloodstream.

The mucosal surfaces on the upper respiratory tract are protected by a highly specialized epithelial barrier. Mucin is a defense mechanism against respiratory infection (5–8), and *S. pneumoniae* must encounter mucins during colonization. The mucin superfamily is classified into two categories, namely, MUC designating human genes and Muc designating mouse genes (5, 9, 10). Gel-forming secreted mucins (e.g., MUC5AC and MUC5B) play a critical role in host defense (11–14), but transmembrane mucins (e.g., MUC1, MUC4, and MUC16) have distinct functions. These mucins form a dense glycocalyx on the apical surface of epithelial cells, and they serve as a physical and biochemical barrier against microbial adhesion and invasion. Previous studies examining cigarette smoke-induced mucin responses have largely focused on secreted gel-forming mucins, such as MUC5AC and MUC5B, particularly in respiratory pathogen infection models (15, 16).

MUC16 is a high-molecular weight transmembrane mucin with an extensively glycosylated extracellular domain. The biochemical mechanism of MUC16 cleavage by pneumococcal metalloproteinase has been described (17), but there is not yet a clear understanding of the *in vivo* significance of MUC16 in preventing bacterial dissemination and systemic disease. Specifically, it is unclear whether the absence of MUC16 compromises the host’s defense during the early stages of infection, thereby facilitating progression to bacteremia and sepsis.

Taking into account mucin biology, epithelial barrier integrity, and interactions with environmental factors, we hypothesize that MUC16 may play a key role in preventing IPD. To address this, we used a Muc16 knockout (KO) mouse model to study the development of invasive pneumococcal infections from nasal colonization *in vivo*. We also introduced the short-term cigarette smoke-exposed model, which is one of the representative environmental factors that predisposes pneumococcal infection by impairing epithelial barrier integrity (2, 4), to highlight mucosal barrier impairment caused by Muc16 deficiency. Because epithelial barrier molecules often become critical only under environmental stress conditions, we incorporated a cigarette smoke extract (CSE) exposure model to examine whether Muc16 becomes functionally important when mucosal integrity is compromised. We aim to define the functional contribution of MUC16 to mucosal defense and sepsis progression during pneumococcal infection.

## RESULTS

### Muc16 was expressed on the mucosal surface of the olfactory epithelium

**Fig 1:**
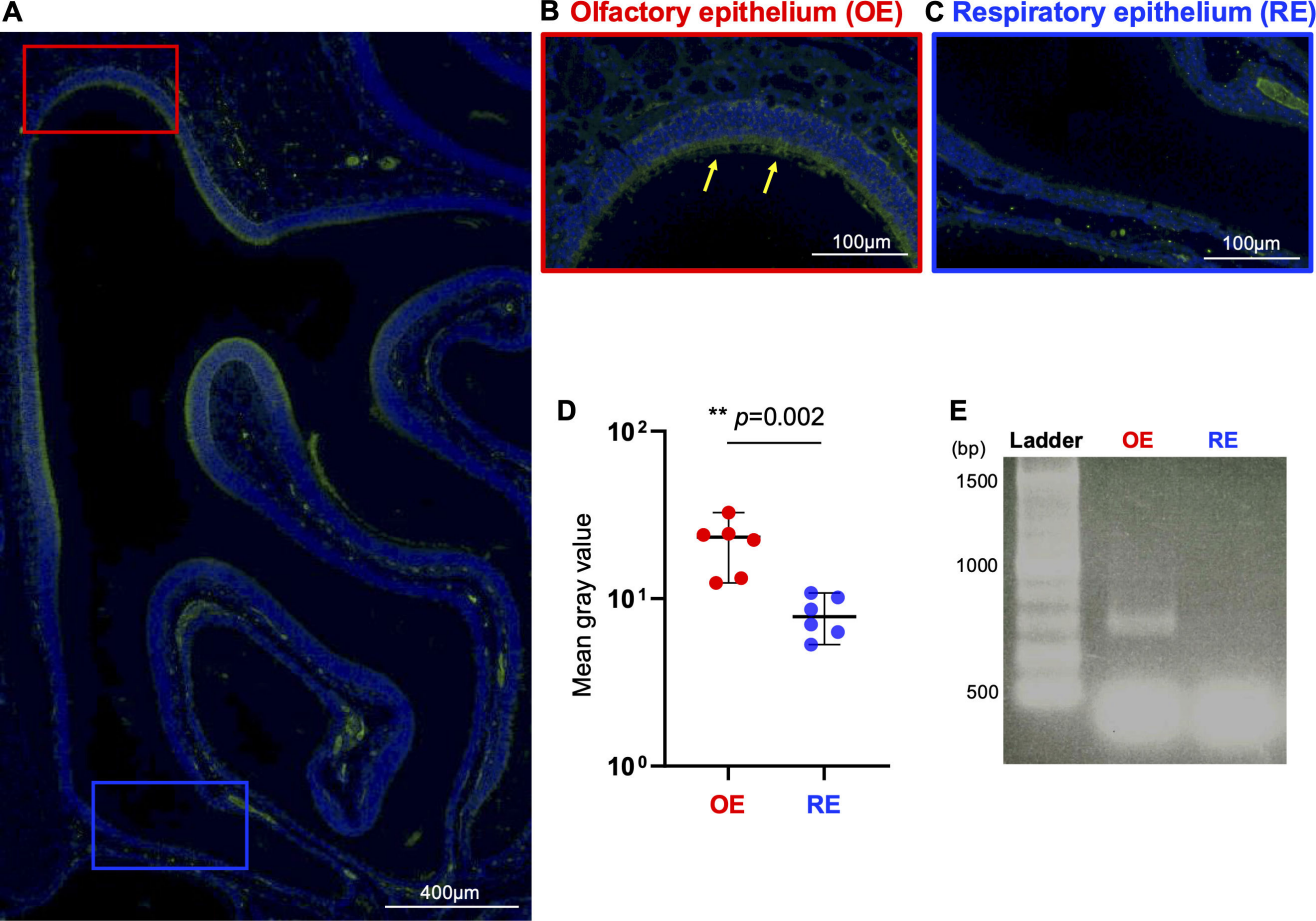
Expression of Muc16 in nasal epithelium. (**A**) Representative image of immunofluorescence staining for Muc16 (green) in nasal cavity. Yellow arrows indicate the expression of Muc16. The upper red box shows olfactory epithelium (OE) (**B**), and the lower blue box shows respiratory epithelium (RE) (**C**). (**D**) Muc16 fluorescence intensity in OE and RE. Mean gray values were obtained from both left and right sides of OE and RE in mice (*n* = 3) from three independent experiments and compared using Mann-Whitney *U* test. A *P* value < 0.05 was considered statistically significant. (**E**) RT-PCR for Muc16 gene transcript of OE and RE.

The expression and localization of Muc16 in the murine nasal cavity were confirmed through both histological and molecular analyses. To determine whether Muc16 is positioned at anatomical sites relevant to pneumococcal entry and mucosal barrier defense, we first examined the localization of Muc16 in the nasal epithelium. Immunofluorescent staining demonstrated the expression of Muc16 in the nasal cavity (Fig. 1A). The olfactory epithelium (OE) is localized in the dorsal-posterior region, particularly along the nasal septum and the superior nasal conchae. In contrast, the respiratory epithelium (RE) is predominantly found in the ventral-anterior region, which covers the inferior nasal conchae and the lateral walls of the nasal cavity. Muc16 was predominantly localized in the OE (Fig. 1B), but not in RE (Fig. 1C). Quantitative analysis of Muc16 signal showed significantly stronger intensity in the OE than the RE (Fig. 1D). In reverse transcription-polymerase chain reaction (RT-PCR) analysis, messenger RNA of Muc16 was also expressed in the OE but not in the RE (Fig. 1E).

### Muc16 deficiency worsened invasive pneumococcal infection

**Fig 2:**
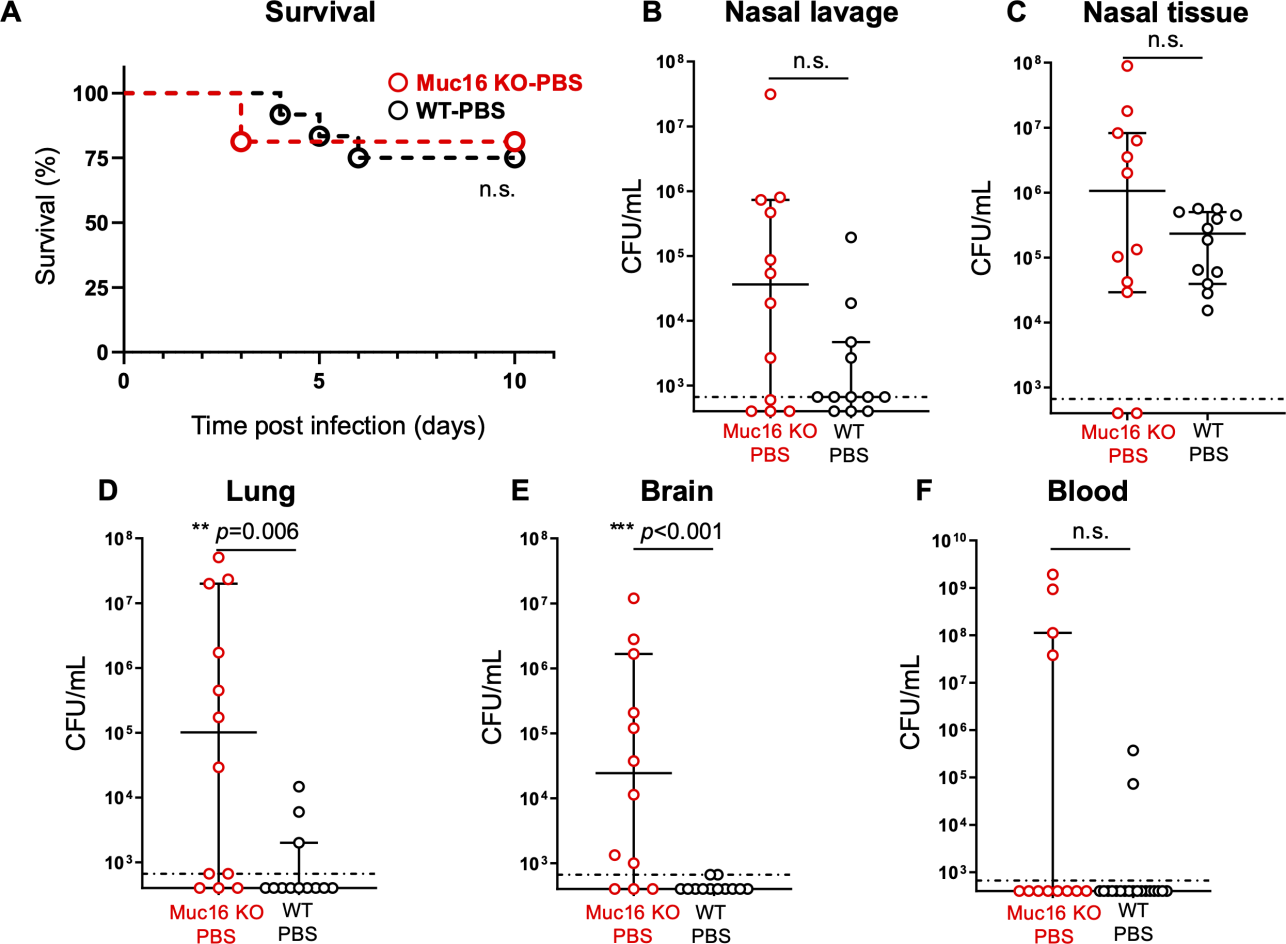
Survival and pneumococcal amounts following intranasal pneumococcal infection in PBS-pretreated mice. (**A**) Survival curves following intranasal pneumococcal infections. Red and black lines indicate Muc16 KO (*n* = 17) and WT mice (*n* = 15), respectively. (**B–F**) Pneumococcal amount in nasal lavage (**B**), nasal tissue (**C**), lung (**D**), brain (**E**), and blood (**F**). Red and black opened circles indicate Muc16 KO (*n* = 12) and WT mice (*n* = 12 in nasal lavage, nasal tissue, lung, and brain, and 19 in blood), respectively. Data represent pooled data from three independent experiments for each group. Survival curves were analyzed using the log-rank test. The pneumococcal amounts were compared using the Mann-Whitney *U* test. A *P* value < 0.05 was considered statistically significant. n.s. indicates not significant.

In the phosphate-buffered saline (PBS)-pretreated group, there were no significant differences in the time course of survival following nasal pneumococcal challenge between Muc16 KO and wild-type (WT) mice (Fig. 2A). Although pneumococci were not detected in nasal lavage samples from some mice in both groups (Fig. 2B), *S. pneumoniae* was detected at comparable levels in nasal tissues from almost all WT and Muc16 KO mice, indicating that nasal colonization was successfully established in both groups (Fig. 2C). However, the numbers of pneumococci in the lungs and brain were significantly higher in Muc16 KO mice than in WT mice (Fig. 2C and D). There was no significant difference in the number of pneumococci in the blood between Muc16 KO and WT mice (Fig. 2E).

### Muc16 deficiency synergistically exacerbates mortality following nasal pneumococcal challenge under cigarette smoke extract exposure

**Fig 3:**
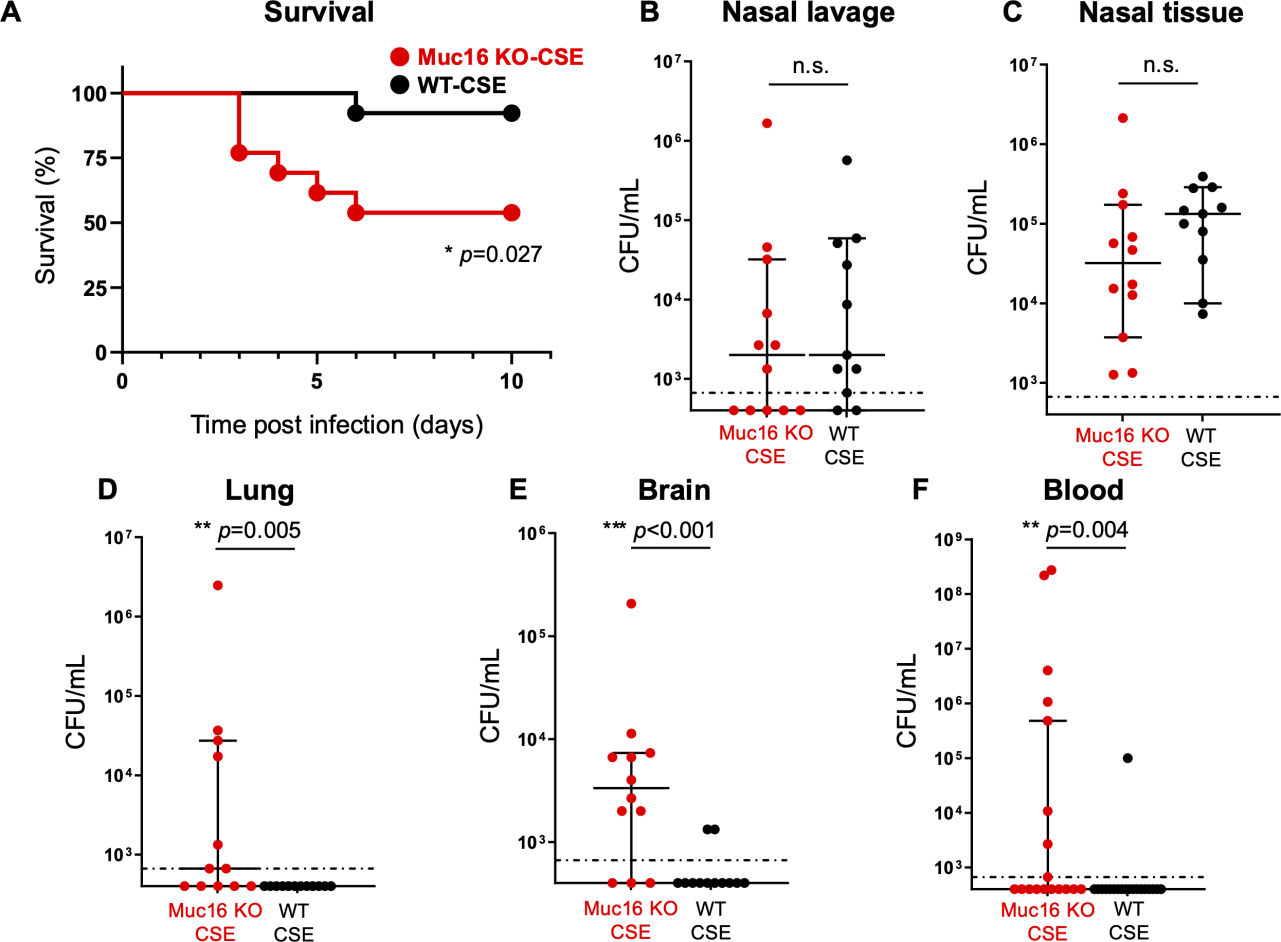
Survival and pneumococcal amounts following intranasal pneumococcal infection in CSE-pretreated mice. (**A**) Survival curves after intranasal infection. Red and black lines indicate Muc16 KO (*n* = 15) and WT mice (*n* = 15). (**B–F**) Pneumococcal amount in nasal lavage (**B**), nasal tissue (**C**), lungs (**D**), brain (**E**), and blood (**F**). Red and black closed circles indicate Muc16 KO (*n* = 12 in nasal lavage, nasal tissue, lungs, and brain, and 18 in blood) and WT mice (*n* = 11 in nasal lavage and nasal tissue, 12 in lungs and brain, and 19 in blood), respectively. Data represent pooled data from three independent experiments for each group. Survival curves were analyzed by the log-rank test. The pneumococcal amounts were compared using the Mann-Whitney *U* test. A *P* value < 0.05 was considered statistically significant. n.s. indicates not significant.

In the group pretreated with CSE, Muc16 KO mice exhibited significantly poorer survival than WT mice (Fig. 3A). However, the numbers of pneumococci in nasal lavage and tissue were not significantly different between Muc16 KO and WT mice (Fig. 3B and C). In contrast, the numbers of pneumococci in the lungs, brain, and blood were significantly higher in Muc16 KO mice than in WT mice (Fig. 3C through E).

Overall, Muc16 did not appear to influence pneumococcal colonization in the nose, but it did appear to play an important role in limiting bacterial invasion in the CSE-pretreated group.

### Muc16 deficiency was associated with elevated systemic IL-6 levels

**Fig 4:**
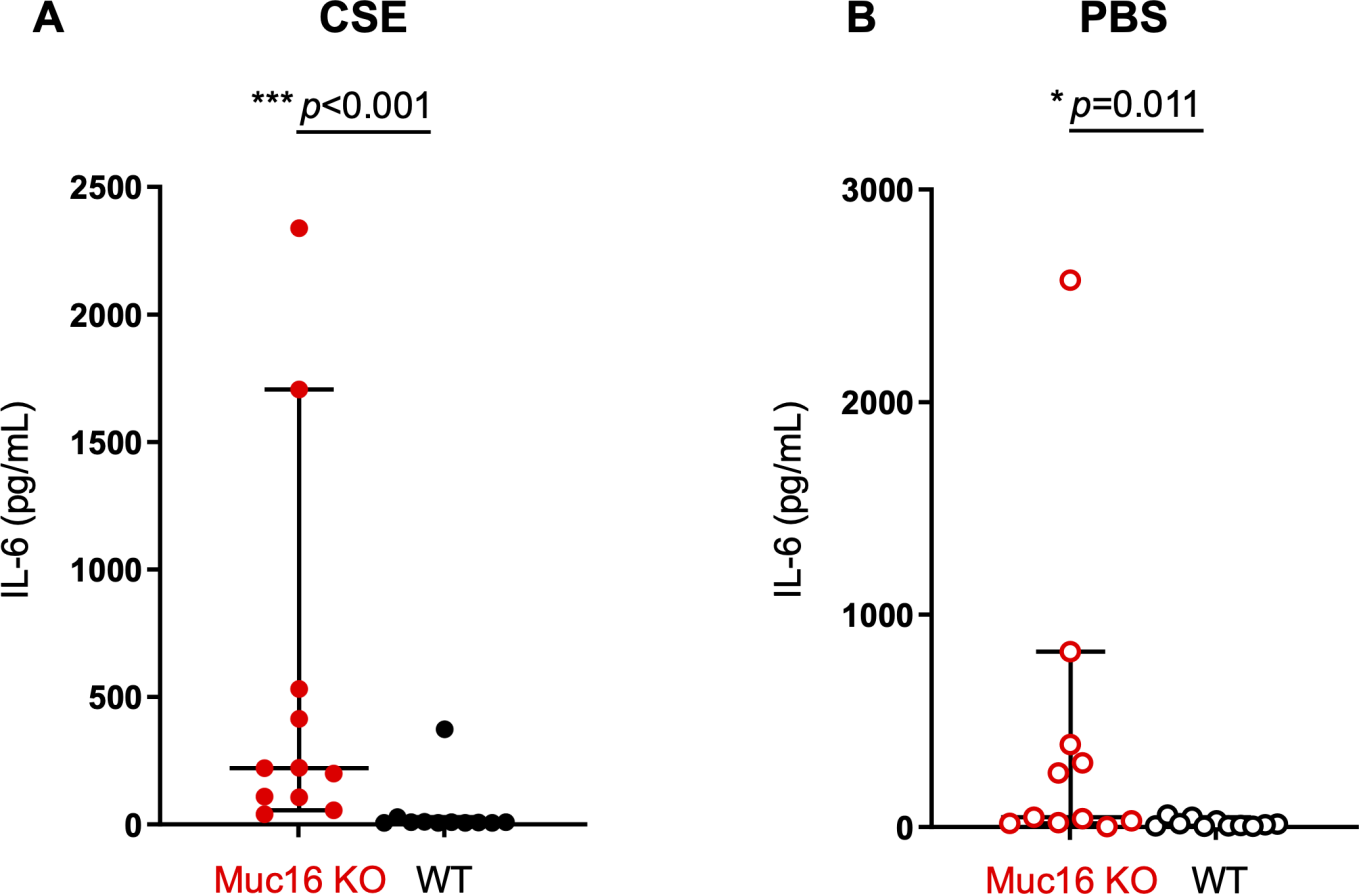
Serum cytokine levels. Serum IL-6 levels were measured by ELISA. (**A**) CSE-pretreated group. Red closed circles represent Muc16 KO mice (*n* = 11), and black closed circles represent WT mice (*n* = 11). (**B**) PBS-pretreated group. Red opened circles represent Muc16 KO mice (*n* = 11), and black opened circles represent WT mice (*n* = 11). Data represent pooled data from two independent experiments for each group. Statistical analysis was performed using the Mann-Whitney *U* test. A *P* value < 0.05 was considered statistically significant.

In both CSE and PBS-pretreated groups, the levels of IL-6 in blood were significantly elevated in Muc16 KO mice compared with in WT mice (Fig. 4A and B). In contrast, no significant differences were observed in the mRNA expression levels of CXCL-1, CXCL-2, TNF-α, and IL-6 in nasal lavage between WT and Muc16 KO mice (Fig. S1A through H).

### Migration of polymorphonuclear leukocytes was suppressed by deficiency of Muc16

**Fig 5:**
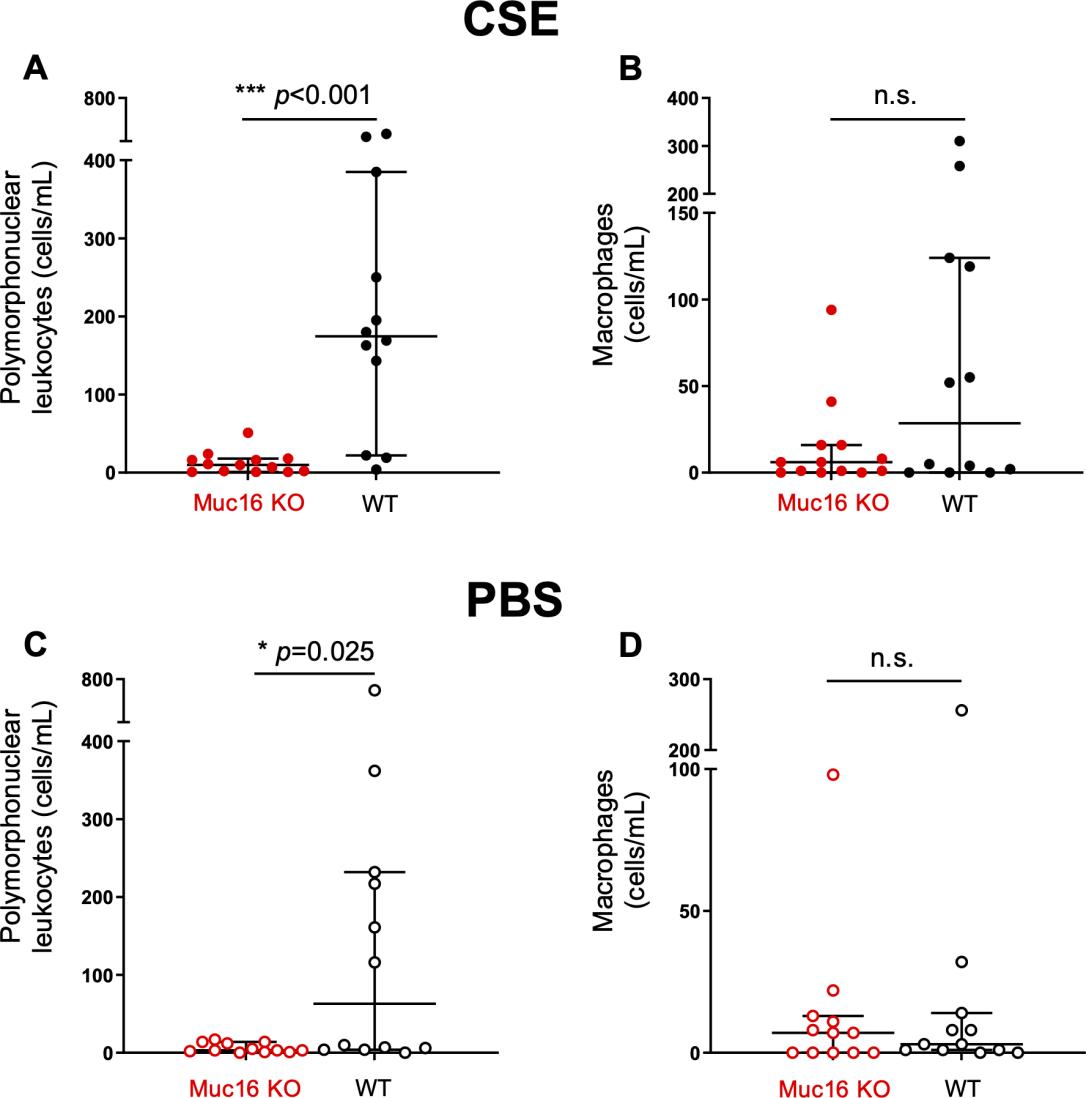
Inflammatory responses in the nasal cavity. Inflammatory cell populations in the nasal cavity in the CSE-pretreated group (**A and B**) and in the PBS-pretreated group (**C and D**). Polymorphonuclear leukocytes (**A and C**) and macrophages (**B and D**) in nasal lavages were evaluated by flow cytometry. Red closed circles represent Muc16 KO mice (*n* = 13), and black closed circles represent WT mice (*n* = 12) in the CSE-pretreated group. Red opened circles represent Muc16 KO mice (*n* = 12), and black opened circles represent WT mice (*n* = 12) in the PBS-pretreated group. Data represent pooled data from three independent experiments for each group. Statistical analysis was performed using the Mann-Whitney *U* test. A *P* value < 0.05 was considered statistically significant. n.s. indicates not significant.

In both CSE and PBS-pretreated groups, the numbers of polymorphonuclear leukocytes in nasal lavage were significantly lower in Muc16 KO mice than in WT mice, whereas there was no apparent difference in the numbers of macrophages (Fig. 5A through D).

### Deficiency of Muc16 disrupted mucosal epithelium

**Fig 6:**
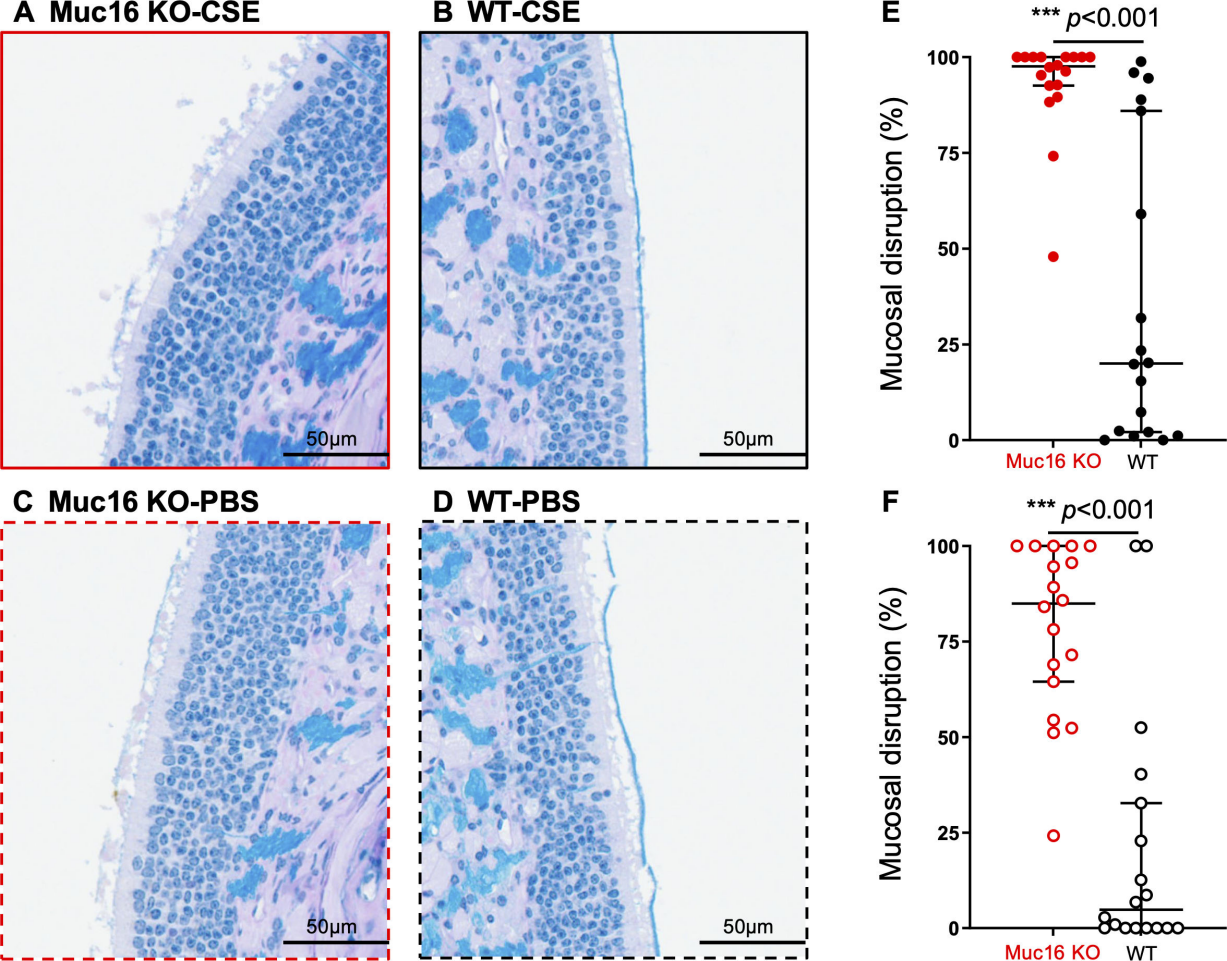
Histological analysis of mucus and pneumococcus. Representative images of double staining of nasal cavity by Alcian blue-periodic acid Schiff and immunohistochemistry against type 6A pneumococcus in CSE- (**A and B**) and PBS-pretreated groups (**C and D**). (**A and C**) Muc16 KO mice. (**B and D**) WT mice. (**E and F**) Proportion of mucosal disruption. CSE- (**E**) and PBS-pretreated groups (**F**). Percentage of mucosal disruption was obtained from both left and right sides of OE at three different levels of nasal septum in mice (*N* = 3) from three independent experiments and compared using Mann-Whitney *U* test. Red closed circles represent Muc16 KO mice (*n* = 18), and black closed circles represent WT mice (*n* = 18) in the CSE-pretreated group. Red opened circles represent Muc16 KO mice (*n* = 18), and black opened circles represent WT mice (*n* = 18) in the PBS-pretreated group. A *P* value < 0.05 was considered statistically significant.

**Fig 7:**
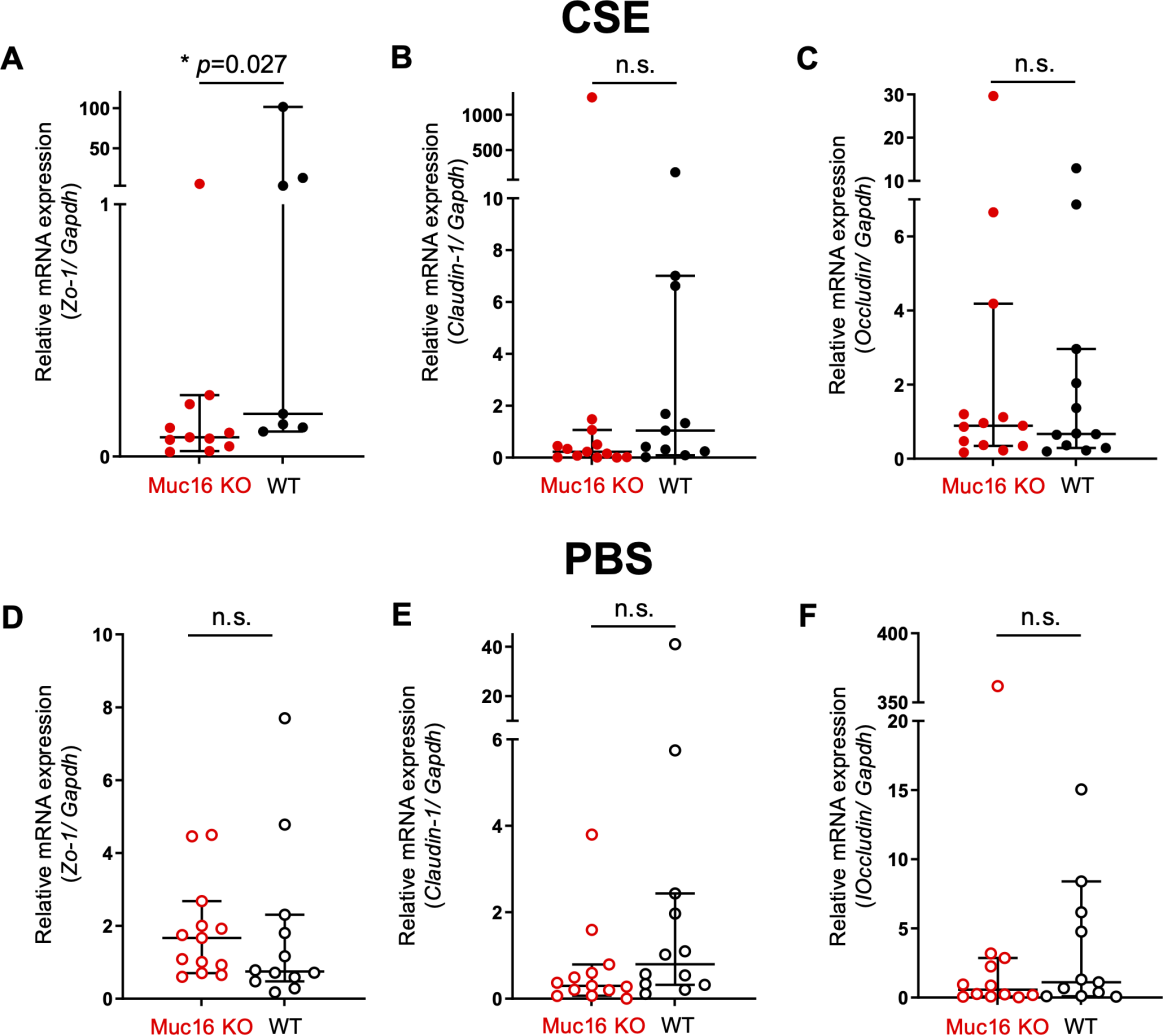
Expression of tight junction molecules. (**A–C**) CSE- and (**D–F**) PBS-pretreated groups. (**A and D**) *Zo-1*, (**B and E**) *Claudin-1*, and (**C and F**) *Occludin*. Expression of the target gene was adjusted to *glyceraldehyde-3-phosphate dehydrogenase* (GAPDH) and indicated as the fold change over the WT mice. Red closed circles represent Muc16 KO mice (*n* = 11 in *Zo-1*, 13 in *Claudin-1* and 13 in *Occludin*), and black closed circles represent WT mice (*n* = 7 in *Zo-1*, 11 in *Claudin-1* and 12 in *Occludin*) in the CSE-pretreated group. Red opened circles represent Muc16 KO mice (*n* = 13 in *Zo-1*, 12 in *Claudin-1* and 12 in *Occludin*), and black opened circles represent WT mice (*n* = 12 in *Zo-1*, 11 in *Claudin-1* and 11 in *Occludin*) in the PBS-pretreated group. Data represent pooled data from three independent experiments for each group. Mann-Whitney *U* test was used for the statistical analysis. A *P* value < 0.05 was considered statistically significant. n.s. indicates not significant.

In Muc16 KO mice, mucosal alignment of the nasal epithelial surface was disrupted in both CSE- and PBS-pretreated groups (Fig. 6A through D). The proportions of the disrupted mucosa in the OE were significantly increased in Muc16 KO mice compared to WT mice in both CSE- and PBS-pretreated group (Fig. 6E and F). There was no significant difference in pneumococcal colonization. Regarding the expressions of the tight junction molecules, *Zo-1* was significantly suppressed in Muc16 KO mice compared with in WT mice in the CSE-pretreated group (Fig. 7A). In contrast, there were no differences in any of the analyzed molecules in the PBS-pretreated group (Fig. 7B through F).

## DISCUSSION

Muc16 was shown here in a mouse model to have important roles in preventing IPD. It plays a dual role in modulating the infiltration of inflammatory cells and in maintaining mucosal barrier in nasal mucosa. Notably, Muc16 deficiency significantly reduced survival following invasive infection and downregulated the expression of tight junction molecule in the CSE-pretreated group. The importance of Muc16 in host defense is thought to be highlighted by these findings, particularly when the mucosa is compromised by environmental stress, such as cigarette smoke exposure.

Mucosal epithelium is essential for defense against infections, and its maintenance is important for protection against pathogens (18). Muc16 has been shown to contribute to epithelial homeostasis in the cornea (6), but its localization and function have been unclear in the upper respiratory tract. MUC16 was reported to be expressed in human nasal mucosa and said to be involved in physiologic mucin production, but was not related to nasal mucosal inflammation (5). Our findings further revealed that Muc16 is more abundantly expressed in the OE than in the RE, exhibiting a localization pattern similar to that of Muc5ac and Muc5b in murine nasal cavity (19). The OE has been shown to have an important role in the prevention of nose-to-brain translocation of infections (20–22). Preservation of the OE against environmental damages, including cigarette smoke, is, therefore, thought to be an important aspect of the mucosal defense mechanism (23).

Recent studies have highlighted the OE as a critical immunological interface that regulates pathogen entry at the boundary between the nasal cavity and the central nervous system. The olfactory mucosa is now recognized as an important component of “olfactory barrier immunity,” integrating epithelial barrier integrity, mucosal glycocalyx structures, and local immune responses to restrict microbial invasion toward deeper tissues and the brain (22). Disruption of this barrier has been implicated in the pathogenesis of several respiratory and neuroinvasive infections. In this context, the preferential localization of Muc16 in the OE observed in the present study suggests that transmembrane mucins may represent an important structural component of this barrier system. Loss of Muc16 may, therefore, weaken the glycocalyx layer at this critical interface, facilitating bacterial penetration across the mucosal surface and subsequent systemic dissemination.

The worsened survival of Muc16 KO mice in our CSE-pretreated group may reflect weakened defense in the OE. *S. pneumoniae* was shown in a previous *in vitro* study to secrete a metalloproteinase that selectively cleaves Muc16, thereby disrupting the glycocalyx barrier and enhancing bacterial adherence and invasion at epithelial junctions (17). However, this mechanism has not previously been confirmed *in vivo*. Our *in vivo* findings support this theory, demonstrating that the genetic loss of Muc16 makes hosts more susceptible to systemic infection following increased mucosal disruption under both PBS- and CSE-pretreated conditions.

In Muc16 KO mice, the nasal burden of *S. pneumoniae* was comparable to that in WT mice. In the present study, we used a relatively low inoculum (1 × 10^6^ CFU/mouse) compared with several previously reported pneumococcal colonization models that used higher infection doses (24, 25). This dose was intentionally selected because wild-type mice rarely develop invasive disease at this level of exposure, allowing differences in susceptibility between WT and Muc16 KO mice to be evaluated. Although pneumococci were not detected in nasal lavage samples from some mice, comparable numbers of *S. pneumoniae* were recovered from nasal tissue cultures in almost all mice in both groups, indicating that nasal colonization was successfully established under these experimental conditions.

Muc16 KO mice exhibited widespread dissemination of pneumococci and significantly elevated serum IL-6 levels. Elevated IL-6 levels may primarily reflect increased systemic bacterial dissemination rather than a direct regulatory role of Muc16 in systemic cytokine production. Because pneumococcal dissemination to the brain is closely associated with bacteremia in this model, increased brain CFU likely reflects systemic dissemination rather than a direct effect of Muc16 on brain invasion. Similarly, bacterial counts detected in highly vascularized tissues, such as the lungs or nasal tissue, may partially reflect circulating bacteria during systemic infection rather than true tissue-specific invasion. Therefore, these measurements should be interpreted primarily as indicators of systemic dissemination rather than direct evidence of organ invasion. Muc16 deficiency is suggested by these findings to impair nasal mucosal barrier function and early immune responses, which allows systemic invasion (26). In previous reports (27), MUC16 in the airway has been shown to be predominantly expressed on both apical membrane of ciliated epithelial cells and the ductal epithelium of airway submucosal glands, where its extended, highly glycosylated extracellular domain integrates with the mucous layer to form a protective barrier. In the current study, histopathological evaluation revealed that loss of MUC16 resulted in disruption of the mucous layer, which is considered to facilitate pneumococcal adherence to the airway epithelium and subsequent invasion into deeper tissues (8).

In the early phase of bacterial infection, neutrophils play a critical role in eliminating invading pathogens at the mucosal surface (28, 29). Despite no major differences in pro-inflammatory cytokines or chemokines, there was significant reduction of neutrophil infiltration, which indicates that Muc16 may influence neutrophil recruitment. At present, direct evidence demonstrating that MUC16 itself drives neutrophil migration is still lacking. However, several lines of indirect evidence support a potential role for MUC16 in regulating neutrophil behavior at the epithelial interface. First, recent studies have shown that MUC16 interacts with Siglec-9 on neutrophils, promoting an activated inflammatory phenotype and enhancing the production of IL-8 and CXCL3, chemokines that can secondarily facilitate neutrophil recruitment (30). Second, MUC16 has been identified as a functional selectin ligand, implicating it in the initial adhesion and rolling steps required for neutrophil extravasation into inflamed tissue (31). These findings suggest that MUC16 may contribute to neutrophil recruitment through modulation of epithelial-immune cell interactions and cell-to-cell adhesion dynamics rather than through direct chemotactic signaling. Taken together, our results raise the possibility that loss of MUC16 compromises the physical and molecular interface required for efficient neutrophil accumulation at the mucosal surface during pneumococcal infection. In addition, several neutrophil chemoattractants have been reported to contribute to neutrophil recruitment during pneumococcal infection, including CXCL5, CXCL8, leukotriene B4 (LTB4), and the lipid chemoattractant hepoxilin A3 (32–35). These mediators were not examined in the present study but may represent additional pathways through which epithelial barrier molecules such as Muc16 influence neutrophil migration in the nasal mucosa. Further mechanistic studies, including direct assays of neutrophil migration and adhesion in the presence or absence of MUC16, will be necessary to clarify the precise role of MUC16 in neutrophil recruitment and mucosal host defense.

Tight junction molecules are also important for the epithelial barrier against infection (36). Muc16 contributes to the regulation of cell-to-cell adhesion and tight junction function (7, 37). External factors, such as cigarette smoke, are known to downregulate the expression of tight junction molecules (38). Although the interaction between airway MUC16 and tight junction needs future consideration, the suppressed expression of *Zo-1* in CSE-pretreated group in Muc16 KO mice suggested the vulnerability of mucosal barrier integrity against external factors under Muc16 deficiency. These differential effects on tight junction components suggest that Muc16 deficiency may influence epithelial barrier integrity through context-dependent mechanisms rather than uniformly suppressing all tight junction proteins.

There are differences in background factors and conditions related to CSE exposure and Muc16 KO, so it would perhaps be more appropriate to evaluate the results between pairs of groups with matching conditions than to compare all four groups simultaneously. This approach minimizes the influence of confounding factors and allows us to better understand the role of Muc16 in mucosal defense. However, the differences in sequence, ectodomain size, and expression between human and mouse MUC16/Muc16 make it difficult to infer human MUC16 function from Muc16 KO mice. Therapeutic approaches aimed at preserving mucin integrity may have value in reducing the risk of sepsis.

This study has several limitations. Because this study focused on host responses during pneumococcal infection, uninfected control groups were not included in the current experimental design. Therefore, the baseline effects of Muc16 deficiency or cigarette smoke exposure on epithelial barrier integrity and inflammatory responses in the absence of infection remain to be determined and should be addressed in future studies. In addition, this study focused exclusively on MUC16, although other mucins (e.g., MUC1 and MUC4) may also contribute to barrier function. The precise mechanisms by which cigarette smoke exposure worsens infection outcomes also remain unclear. We applied a previously established short-term cigarette smoke exposure model (2) intended to influence mucosal defense mechanisms. Cigarette smoke disrupts the mucosal barrier and induces local inflammation (2, 39), and it has been shown to increase nasal colonization and the risk of invasive infection in both animal and human studies (4, 40, 41). However, the mechanisms underlying the CSE-specific effects observed under Muc16 deficiency, particularly those related to tight junction regulation, remain to be clarified. Furthermore, future studies should investigate how mucin expression is regulated during infection and whether modulation of MUC16 or its proteolytic cleavage influences infection outcomes.

In conclusion, MUC16 is a key mucosal barrier molecule that restricts pneumococcal dissemination and mitigates the severity of sepsis, particularly under cigarette smoke exposure. Muc16 contributes to mucosal defense by preserving epithelial barrier integrity and supporting immune cell infiltration rather than by reducing bacterial burden. We suggest these findings provide important insight into the barrier system of transmembrane mucin for the preventive strategy of invasive pneumococcal infection.

## MATERIALS AND METHODS

### Bacterial strain and growth conditions

P2431, a streptomycin-resistant *S. pneumoniae* serotype 6A strain (kindly provided by Prof. Jeffrey N. Weiser, New York University), which can cause bacteremia without pneumonia, was used in all experiments (2, 25). *S. pneumoniae* P2431 was grown in tryptic soy broth (Becton Dickinson, Franklin Lakes, NJ) at 37°C until the mid-exponential phase. When the bacterial culture reached the desired optical density at 600 nm, the bacteria were washed and diluted in sterile PBS for inoculation. Bacterial stocks were stored in 20% glycerol at −80°C until the experiments. Quantitative culture was performed by plating 10-fold serial dilutions in triplicate on tryptic soy agar plates containing streptomycin (200 µg/mL) and catalase (6300 U/plate) (Worthington Biochemical Corporation, Lakewood, NJ). Plates were incubated overnight at 37°C under 5% CO_2_ condition.

### Mice

The mice used in this experiment were approximately 6 weeks old. C57BL/6J WT mice (Japan SLC, Hamamatsu, Japan) and Muc16 KO mice of C57BL/6J background (Jackson Laboratory, Bar Harbor, ME, USA) were used (6, 42). All mice were maintained in a conventional animal facility at Wakayama Medical University.

### Cigarette smoke extract

Cigarette smoke extract (CSE) was obtained from CMIC Pharma Science Co., Ltd. (Yamanashi, Japan). Briefly, CSE is prepared by bubbling a stream of smoke from 50 of Hi-Light cigarettes (Japan Tobacco Inc., Tokyo) through 50 mL of saline (43). For standardization, CSE was diluted 100-fold, and an OD at 267 nm was used as the indicator of concentration; the standardized concentration was adjusted to ~ 1.308. Undiluted CSE was used for all experiments similarly as previous reports (2, 23, 43). CSE was stored at −80°C until use.

### Mouse model of cigarette smoke exposure and infection

From experimental days −4 to −1, mice were intranasally given 10 µL of CSE or PBS twice a day without anesthesia. On experimental day 0, mice were intranasally inoculated with 1.0 × 10^6^ CFU of *S. pneumoniae* suspended in 10 µL of PBS without anesthesia. The mice were euthanized by isoflurane asphyxiation for sampling on experimental day 2 or monitored until showing signs of lethal infection, including sepsis and sustained bacteremia (decreased motor activity, shivering, and messy fur). Approximately 500 µL of blood was collected by cardiac puncture from each mouse. Next, both lungs and the entire brain were obtained and homogenized in 1 mL of sterile PBS. Then, the upper respiratory tract was lavaged with 200 µL of sterile PBS from a 25 G needle inserted into the trachea, and lavages were collected from the nares (2, 25, 44). After washing the nasal cavity, the nasal tissue without brain was homogenized in 1 mL of sterile PBS. The nasal lavages, homogenized tissues, or blood were serially diluted and plated on tryptic soy agar plates containing streptomycin (200 µg/mL) and catalase (6,300 U/plate) and incubated overnight at 37°C under 5% CO_2_. The pneumococcal colonies were counted to determine the colonization in each mouse. The lower limit of detection was 666 CFU/mL.

### Histological analysis

Whole skulls were fixed in 4% paraformaldehyde for 2 days, and then decalcified with ethylenediaminetetraacetic acid for 2 weeks. Nasal tissues were sectioned coronally at the nasal cavity. For immunofluorescent for Muc16, anti-Muc16 primary antibody was used (Santa Cruz Biotechnology, Inc., Dallas, TX). For visualizing mucus and pneumococcus, double staining of Alcian blue-periodic acid Schiff (AB-PAS) and immunohistochemistry were performed using rabbit antiserum against type 6A pneumococcal polysaccharide (Statens Serum Institut, Denmark). Images were captured using NDP.view2 (Hamamatsu Electronic Press Co., Hamamatsu, Japan).

### Fluorescence intensity quantification of Muc16

Quantitative analysis of Muc16 signal intensity was performed at ×200 magnification on both the left and right sides of the coronal section of OE in the olfactory cleft and RE in the nasal floor. Signal intensity was quantified as the mean gray value using ImageJ software (National Institutes of Health, Bethesda, MD). These quantitative values were used for statistical comparisons between OE and RE.

### Calculation of the proportion of mucosal disruption

Disrupted mucosa of the OE on the nasal septum was performed at ×400 magnification on both the left and right sides of the coronal section stained by AB-PAS staining at three different levels. The levels were 250, 500, and 750 μm from the upper end of the nasal cavity. These regions were chosen because these areas contain the OE (45) and are nearly straight, making them easy to measure for length. The measurement of disrupted mucosa was performed as shown in Fig. S2 using ImageJ software. Briefly, length of OE was measured at ×400 magnification, and length of disrupted mucosa was calculated by summing the total length of epithelial regions with mucus layer defects observed in AB-PAS staining. The proportion of disrupted mucosa was calculated by dividing the total sum of the length of disrupted mucosa by the length of the OE.

### Reverse transcription polymerase chain reaction (RT-PCR) analysis of *Muc16* gene transcripts

RNA was extracted from OE and RE of wild-type mice using mirVANA miRNA Isolation Kit (Thermo Fisher Scientific, Waltham, MA). Based on the previous literature, RE was collected from the anterior portion of the incisive papilla and the ventral part of the nasal cavity, while OE was collected from the posterior portion and the dorsal part of the nasal cavity (19). cDNA was synthesized with a High-Capacity cDNA Reverse Transcription Kit (Thermo Fisher Scientific). The transcripts of *Muc16* were analyzed by PCR using GoTaq Green Master Mix (PROMEGA, Madison, WI). Primers were purchased from Integrated DNA Technologies (Singapore) (Table 1).

**TABLE 1:** Summary of PCR primer sequences

Gene	Forward primer (5′−3′)	Reverse primer (5′−3′)
MUC16	GCC CAT GTT CAA GAA TAG CAG TAT TGG	GTA GCA GAG AGG GGC TTG TGG TTG
ZO-1	GGA GCT ACG CTT GCC ACA CT	GGT CAA TCA GGA CAG AAA CAC AGT
Claudin-1	TCT ACG AGG GAC TGT GGA TG	TCA GAT TCA GCA AGG AGT CG
Occludin	AGA GGC TAT GGG ACA GGG CTC TTT GG	CCA ACA GGA AGC CTT TGG CTG CTC TTG G
CXCL1	CTG GGA TTC ACC TCA AGA ACA TC	CAG GGT CAA GGC AAG CCT C
CXCL2	CCA CCA ACC ACC AGG CTA C	GCT TCA GGG TCA AGG GCA AA
TNF-α	TGT GCC TCA GCC TCT TCT C	GAG CCC ATT TGG GAA CTT CT
IL-6	AGT TGC CTT CTT GGG ACT GA	TCC ACG ATT TCC CAG AGA AC
GAPDH	AGG TCG GTG TGA ACG GAT TTG	TGT AGA CCA TGT AGT TGA GGT CA

### Quantitative RT-PCR (qRT-PCR)

After the collection of nasal lavages by PBS, nasal washes were performed with RLT lysis buffer. RNA was isolated using RNeasy Mini Kit (Qiagen, Mississauga, ON, Canada) with RNase-Free DNase I and DNase 10× Reaction Buffer (Promega). cDNA was synthesized as described above. The relative gene expressions of CXCL-1, CXCL-2, tumor necrosis factor (TNF)-α, interleukin (IL)-6, zonula occludens (ZO)-1, claudin-1, and occludin were evaluated by qRT-PCR. Primer sequences are summarized in Table 1. qRT-PCR was performed using PowerUP SYBR Green Master Mix (Thermo Fisher Scientific). Gene expression was determined using the ddCT method. Target gene expression was normalized to glyceraldehyde-3-phosphate dehydrogenase (GAPDH) and expressed as the fold change over the WT control group.

### Enzyme-linked immunosorbent assay

Blood samples were centrifuged at 5,000 rpm for 5 min, and the sera were collected for enzyme-linked immunosorbent assay. Quantitative evaluation of IL-6 in sera was performed according to the manufacturer’s protocol (ProteinTech, Rosemont, IL).

### Flow cytometry

Neutrophils (polymorphonuclear cells) in nasal lavages were stained as previously described (2). Pellets of nasal lavages were resuspended in PBS with 1% bovine serum albumin. Cells were stained with a 1:150 dilution of antibodies anti-CD11b-V450 (BD), anti-Ly6G-PerCP-Cy (BD), and anti-CD45-APC-Cy7 (BD) for 30 min on ice in the dark after FcR blocking with a 1:200 dilution of anti-CD16/32 (BioLegend) for 15 min on ice. Cells were then fixed with 4% paraformaldehyde until analysis on FACS Verse (BD). Polymorphonuclear leukocytes were detected as CD11b^+^, Ly-6G^+^, and CD45^+^ events, and macrophages were detected as CD11b^+^, Ly6G^−^, and F4/80^+^ events.

### Statistical analyses

All statistical analyses were performed with GraphPad Prism 10 (GraphPad Software, Inc., San Diego, CA). Differences were determined by Mann-Whitney *U* test. Survival curves were analyzed using log-rank test. *P* values < 0.05 were considered to be statistically significant.

## SUPPLEMENTAL MATERIAL

Figures S1 and S2 spectrum.00824-26-s0001.docx Figure S1: Pro-inflammatory cytokines and chemokines; Figure S2: Calculation of mucosal disruption.

ASM does not own the copyrights to Supplemental Material that may be linked to, or accessed through, an article. The authors have granted ASM a non-exclusive, world-wide license to publish the Supplemental Material files. Please contact the corresponding author directly for reuse.
