# Supplementary material for: Muc16 contributes to protection against invasive pneumococcal infection originating from nasal colonization
Source: Microbiol Spectr. 2026 Jun 26;14(8):e00824-26. doi: 10.1128/spectrum.00824-26 (PMC13435705; doi:10.1128/spectrum.00824-26)
Supplement: Supplementary file 1 [file spectrum.00824-26-s0001.docx]

**Supplemental figure 1. Pro-inflammatory cytokines and chemokines.**

(A-D) CSE-pretreated group, (E-H) PBS-pretreated group. (A and E) *Cxcl-1*, (B and F) *Cxcl-2*, (C and G) *Tnf-α*, (D and H) *Il-6*. Expression of mRNA were determined using ddCT method. Expression of the target gene was adjusted to *glyceraldehyde-3-phosphate dehydrogenase* (*Gapdh)* and indicated as the fold change over the WT group. Red closed circles represent Muc16 KO mice (n=11 in *Cxcl-1* and *Cxcl-2*, 10 in *Tnf-α* and 8 in *Il-6*) and black closed circles represent WT mice (n=7 in *Cxcl-1*, *Cxcl-2* and *Il-6*, and 8 in *Tnf-α*) in the CSE-pretreated group. Red opened circles represent Muc16 KO mice (n=12 in *Cxcl-1* and *Cxcl-2*, 8 in *Tnf-α* and 6 in *Il-6*) and black opened circles represent WT mice (n=11 in *Cxcl-1*, 10 in *Cxcl-2*, 9 in *Tnf-α* and 7 in *Il-6*) in the PBS-pretreated group. Data represent pooled data from three independent experiments for each group. Mann-Whitney U test was used for the statistical analysis. A *p*-value <0.05 was considered statistically significant. n.s. indicates not significant.

**Supplemental figure 2. Calculation of mucosal disruption.**

At ×400 magnification in AB-PAS staining, length of OE was measured as a green line. Length of disrupted mucosa was measured as epithelial regions with mucus layer defects as red lines. Proportion of mucosal disruption was calculated by dividing the total sum of the length of disrupted mucosa by the length of the OE.
